# Isolation and Characterization of Human Synovial Fluid-Derived Mesenchymal Stromal Cells from Popliteal Cyst

**DOI:** 10.1155/2020/7416493

**Published:** 2020-09-18

**Authors:** Fang Li, Jianglin Chen, Mengjia Gong, Yang Bi, Chengchen Hu, Yuanyuan Zhang, Ming Li

**Affiliations:** ^1^Department of Orthopedics, Ministry of Education Key Laboratory of Child Development and Disorders, National Clinical Research Center for Child Health and Disorders, China International Science and Technology Cooperation Base of Child Development and Critical Disorders, Children's Hospital of Chongqing Medical University, Chongqing, China; ^2^Department of Pediatric Research Institute, Chongqing Key Laboratory of Pediatrics, Children's Hospital of Chongqing Medical University, Chongqing, China

## Abstract

Mesenchymal stem cells (MSCs) are multipotent progenitor cells in adult tissues. The aim of this study is to isolate and identify synovial fluid-derived mesenchymal stromal cells (SF-MSCs) from the popliteal cyst fluid of pediatric patients. SF-MSCs were collected from the popliteal cyst fluid of pediatric patients during cystectomy surgery. After cyst fluid extraction and adherent culturing, in vitro morphology, growth curve, and cell cycle were observed. The expression of stem cell surface markers was analyzed by flow cytometry, and expression of cell marker protein was detected by immunofluorescence. SF-MSCs were cultured in osteogenic, adipogenic, and chondrogenic differentiation medium. The differentiation potential of SF-MSCs was analyzed by alkaline phosphatase (Alizarin Red), Oil Red O, and Alcian blue. Antibody detection of human angiogenesis-related proteins was performed compared with bone marrow mesenchymal stem cells (BM-MSCs). The results show that SF-MSCs from the popliteal cyst fluid of pediatric patients showed a shuttle appearance and logarithmic growth. Flow cytometry analysis revealed that SF-MSCs were negative for hematopoietic lineage markers (CD34, CD45) and positive for MSC markers (CD44, CD73, CD90, and CD105). Interstitial cell marker (vimentin) and myofibroblast-like cell marker alpha-smooth muscle actin (*α*-SMA) were positive. These cells could differentiate into osteogenic, adipogenic, and chondrogenic lineages, respectively. Several types of human angiogenesis-related proteins were detected in the cell secretory fluid. These results show that we successfully obtained SF-MSCs from the popliteal cyst fluid of pediatric patients, which have the potential to be a valuable source of MSCs.

## 1. Introduction

Stem cells are capable of self-renewal and multidifferentiation. Stem cells are divided into embryonic and adult stem cells, according to their developmental stage. Embryonic stem cells can be induced to differentiate into almost any type of cell [1]. However, due to immunological rejection and ethical problems, the clinical applications of embryonic stem cells are limited. Adult stem cells are undifferentiated cells within differentiated tissues. Adult stem cells can self-renew and specialize to form tissue-specific cells [2]. Mesenchymal stem cells (MSCs) are attractive as a cell source for regenerative medicine, particularly in the treatment of cartilage injuries [3] and diseases such as osteoarthritis (OA) [4] owing to their potential to repair cartilage. MSCs can be isolated from several different sources, including the bone marrow, adipose tissue, skeletal muscle [5], periosteum [6], and synovium [7]. They have a capacity to attach to cell culture-grade plastic, strong ability to generate colonies, and trilineage potential to become bone, fat, and cartilage cells [8]. In spite of having many characteristics in common, MSCs are influenced by the tissue microenvironment in which they reside, and as a result, MSCs from different tissues present specific traits which serve to distinguish them from one another [9, 10]. For example, MSCs isolated from articulating joints have shown a superior capacity to contribute to cartilage repair [11].

De Bari et al. [7] were the first to isolate synovial fluid-derived mesenchymal stromal cells (SF-MSCs) from knee joints of adult human donors in 2001. Since then, many considerable efforts have been made to investigate the potentiality of using SF-MSCs for tissue engineering of cartilage [12–17]. SF-MSCs are derived from the synovial membrane but exist in the lubricating fluid contained within the joint cavity [18]. Interestingly, it has been reported that both the synovium and cartilage are proven to be derived from a common pool of progenitor cells [19]. What is more, SF-MSCs have shown a stronger ability to produce cartilage cells and have stronger colony formation and amplification capabilities compared with other evaluated MSC types, including those from the periosteum, adipose tissue, and synovial membrane [20, 21].

Synovial mesenchymal stromal cells are widely derived from the synovium, periosteum, tendons, ligaments, synovial fluid, and other sources [6]. The traditional methods for collecting synovial mesenchymal stromal cells include incising synovial tissue or intra-articular injection of digestion factors. However, synovial tissue excision surgery causes considerable trauma to patients, and intra-articular injection or digestion methods can injure the cells. Therefore, SF-MSCs have not been widely collected and used for transplantation. A new approach for collecting SF-MSCs needs to be explored. Popliteal cysts are a common disease in children and require surgery. In this study, we report the isolation and characterization of human SF-MSCs derived from inpatient children with popliteal cysts. We successfully turned waste material into treasure and explored a convenient and efficient way to obtain SF-MSCs from the popliteal cyst fluid of pediatric patients. These cells showed a shuttle appearance and logarithmic growth and maintain high clonogenicity during long-term in vitro culture. Besides, they expressed SF-MSC surface markers (CD44, CD73, CD90, and CD105), interstitial cell markers (vimentin), and alpha-smooth muscle actin (*α*-SMA). They have the potential for multilineage differentiation (osteogenic, chondrogenic, and adipogenic differentiation capacity). We hypothesized that SF-MSCs isolated from popliteal cysts may be useful as a good resource for cartilage regeneration engineering.

## 2. Materials and Methods

### 2.1. Patient Selection, Fluid Collection, and Isolation of Cells

**Figure 1:**
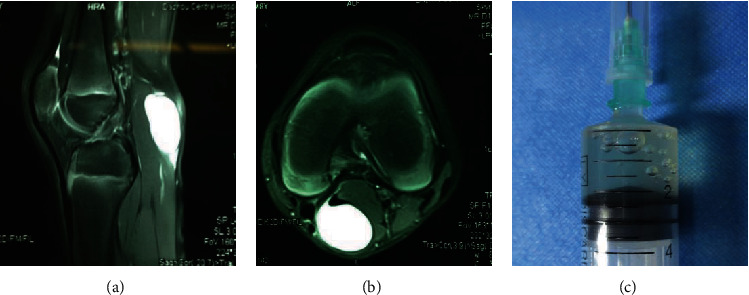
(a) T2-weighted sagittal scan and (b) T2-weighted axial scan of the right knee showing a cystic lesion between the semimembranous muscle and the medial head of the gastrocnemius. The popliteal cyst is filled with liquid. (c) Extraction fluid of popliteal cyst: it shows a colorless mucous liquid with little blood color.

This protocol was approved by the Institutional Review Board (IRB) of the Chongqing Medical University and performed in accordance with the ethical standards prescribed by the Helsinki Declaration of the World Medical Association. Between July 2017 and September 2019, a total of 16 patients composed of 9 males and 7 females (mean age ± SD; 8.8 ± 2.3 (range 5-13) years) with popliteal cysts were treated with surgical resection. Sixteen donors' samples were randomly assigned to different assays for study, and every sample was used in two different assays. These patients were diagnosed by MRI (Figures 1(a) and 1(b)) and did not have other osteoarticular diseases or infectious diseases. The average duration of symptoms was 13.4 weeks. There was no significant difference between the durations from onset of symptoms to date of surgery in these 16 patients. Cyst fluid samples were extracted with 8# syringes (Figure 1(c)) during the operation and transported to the laboratory at 4°C, within 0.5 h of aspiration. Cyst wall tissue was reserved for further research after the cyst fluid was extracted. In vitro, the cyst fluid was fully mixed with advanced Dulbecco's modified Eagle's medium at a 1 : 5 ratio, using advanced Dulbecco's modified Eagle medium (DMEM) containing 10% fetal bovine serum (GIBCO), 2 mM L-glutamine (GIBCO), 100 U/mL penicillin, and 100 *μ*g/mL streptomycin (GIBCO). The compound liquid was transferred to 24-well tissue culture plates at approximately 1 mL per well. The dishes were incubated at 37°C with 5% humidified CO_2_. After 48 h, the adherent cells were photographed by an electronic camera. After 3-4 days, when cells reached 70-80% confluence, they were washed with PBS twice and detached by incubation with trypsin-EDTA (GIBCO) for 1 min, and fresh complete culture medium was added. Subsequent passages were performed in the same way when cells reached confluence. The cells were expanded in a monolayer for 8-9 passages. During the first 15 days, the culture dishes were observed closely to monitor the morphology of adherent cells by microscopy. In addition, portions of the cells from each sample were expanded for further experimentation. The profiles of synovial fluid samples obtained from the patients with popliteal cysts in this study are shown in Table 1.

### 2.2. Expansion of SF-MSCs

#### 2.2.1. Cell Growth Curve

Cultured cells were harvested at passage 2 and then replated in triplicate in 96-well tissue culture plates at 2,500 cells per well in 200 *μ*L complete culture medium (*n* = 3). The medium was changed every 2 days. Cell proliferation was determined using a Cell Counting Kit-8 (CCK-8) cell proliferation assay kit (Dojindo Molecular Technologies) according to the manufacturer's instructions. Absorbance was read at 450 nM with a uQuant™ plate reader daily for up to 9 days of incubation. The results are expressed as multiples of the initial cell numbers.

#### 2.2.2. Calculation of the Doubling Time

Cultured cells were harvested at passage 1 (*n* = 6) and then replated in duplicate in 12-well tissue culture plates at 1000 cells per well in 2 mL complete culture medium. The medium was changed every 2 days. Cells were routinely subcultured every 3-5 days. The initial number (*N*_*i*_), terminal number (*N*_*f*_) of each generation, and the corresponding culture time (*C*_*t*_) (accurate to half an hour) of each generation were recorded. Calculation formula: PD = ln(*N*_*f*_/*N*_*i*_)/ln(2) and DT = *C*_*t*_/PD (*N*_*f*_: final number of cells, *N*_*i*_: initial number of cells, and *C*_*t*_: culture time).

#### 2.2.3. Apoptosis Assay by Flow Cytometry

Annexin V-fluorescein (FITC) staining and propidium iodide (PI) double staining were performed to measure apoptosis by flow cytometry. Cultured cells were harvested at passage 2 and then replated in triplicate in 6-well culture plates at a density of 5 × 10^4^ cells per well containing 2 mL complete culture medium (*n* = 3). The medium was changed every 2 days. After culture for 3, 7, 9, and 11 days, the cells were harvested. The cells were obtained and resuspended in 195 *μ*L 1× binding buffer containing Annexin V-FITC (5 *μ*L) and PI (10 *μ*L), and the cells were incubated away from light for 20 min. The results were evaluated immediately by flow cytometry (BD FACSCanto).

#### 2.2.4. Hoechst 33342 Staining

This assay is continuous with the apoptosis assay and it shared the same cell sample. After 3, 7, 9, and 11 days of culture, the cells were seeded in 6-well plates containing 2 mL medium. After the culture medium was removed, the cells were fixed in 4% paraformaldehyde for 20 min 4°C and were washed three times with phosphate-buffered saline (PBS). The cell nucleus was stained with 5 *μ*g/mL Hoechst 33342 for 10 min at room temperature in the dark and was washed three times with PBS. Nuclear morphology was observed under fluorescence microscopy (Nikon, Tokyo, Japan) to detect cellular nuclear damage.

### 2.3. Cell Cycle Analysis

A total of 1 × 10^7^ cells at passage 3 were harvested and centrifuged at 1500 rpm at 4°C for 5 min (*n* = 3). The cells were resuspended in 100 *μ*L PBS and slowly placed into Eppendorf tubes with 1 mL cold 75% alcohol, centrifuged at 1000 rpm for 4 min, and washed twice with PBS. After adding 0.5 mL 1 mg/mL propidium iodide (YEASEN), the cells were incubated in the dark for 30 min at 4°C and then measured by a BD™ FACSCalibur.

### 2.4. Cell Surface Marker Analysis

The prevalence of MSC-specific surface antigens was determined by flow cytometry. Total 1 × 10^4^ cells at passage 3 were harvested and resuspended in 500 *μ*L staining buffer (SB; PBS containing 1% FBS) and incubated in the dark for 30 min at 4°C with 20 *μ*g/mL (*n* = 5). Then, the cells were stained with the following specific antihuman antibodies: CD24-FITC, CD-29-PE, CD34-PE, CD44-FITC, CD45-FITC, CD73-PE, CD90-PE, CD105-FITC, CD117-FITC, CD146-PE, CD147-PE, and OCT-4 (BD Pharmingen). Immunoglobulin IgG-FITC and IgG-PE conjugated isotype control antibodies were used to determine background fluorescence. Data were analyzed using a FACSCalibur analytical fluorescence-activated cell sorter.

### 2.5. Immunofluorescence

Cells at passage 3 on glass coverslips were fixed with a 4% paraformaldehyde solution (PFA in 0.1 M NaPP) for 30 min and with acetone for 5 min (*n* = 3). Blocking of nonspecific binding sites was performed using a solution of 10% bovine serum albumin (BSA, Sigma®) in 0.1 M PBS buffer solution. Preparations were incubated with the following primary antibodies at 5°C for 12 h in 1% BSA solution and 0.1 M PBS buffer: mouse anti-human vimentin (1 : 500; Ab137321, Abcam®), rabbit anti-human alpha-SMA (Anti-alpha smooth muscle actin) (1 : 100; Ab5694, Abcam®), rabbit anti-human collagen I (1 : 100; Ab34710, Abcam®), and rabbit anti-human pan keratin (1 : 50; Ab185627, Abcam®). The secondary antibodies used were fluorescein isothiocyanate-conjugated goat anti-rabbit IgG antibody (1 : 100, Abcam®), fluorescein isothiocyanate-conjugated goat anti-rabbit IgM antibody (1 : 200, Becton, Dickinson &Company), and rabbit anti-mouse IgG antibody (1 : 100, EarthOx). Nuclei were stained with 1 *μ*g/mL DAPI (1 : 1000; D9542, Sigma-Aldrich®) for 2 h in a 1% solution of BSA and 0.1 M PBS. After three times of washing steps, the coverslips were embedded on slides with Mowiol® (4-88, Seebio®). All surface marker assays were performed at least 3 times to ensure consistent results.

### 2.6. Multilineage Differentiation In Vitro

Cells were induced towards osteogenic, adipogenic, and chondrogenic cells with commercially available differentiation induction and maintenance medium from OriCell®. Protocols were performed according to the manufacturer's instructions and are shortly described here (*n* = 3).

#### 2.6.1. Osteogenic Differentiation

Osteogenesis was induced in 6-well plates using an osteogenic differentiation medium (OriCell®) containing 100 nM dexamethasone, 10 mM b-glycerophosphate, and 50 mg/mL ascorbate. Cells at passage 3 were plated at a density of 2.0 × 104 cells/cm^2^ with 2 mL of 10% FBS DMEM. After 48 hours, the 10% FBS DMEM was discarded and replaced with osteogenic differentiation medium every three days. Cells were maintained in culture for 28 days. Early osteoinductive differentiation was assayed using alkaline phosphatase (ALP, Sigma Aldrich) staining on day 7, and late osteoinductive differentiation was assayed using Alizarin Red (Sigma Aldrich) staining on day 21.

#### 2.6.2. Adipogenic Differentiation

Adipogenesis was induced in 6-well plates using an adipogenic differentiation medium (OriCell®) containing 100 nM dexamethasone, 200 nM insulin, 0.5 mM isobutyl-methylxanthine, and 50 mM indomethacin. Cells were plated at a density of 2 × 10 4 cells/cm^2^ with 2 mL of 10% FBS DMEM. Complete medium changes were performed every 2-3 days with adipogenic differentiation medium for 21 days. At the end of the adipogenesis, these cultured cells were fixed in 4% PFA and stained with Oil Red O (Cyagen) solution.

#### 2.6.3. Chondrogenic Differentiation

Chondrogenesis was induced using the pellet culture method with differentiation medium (Invitrogen) containing 100 nM dexamethasone, 100 nM sodium pyruvate, and 100 nM proline 10 ng/mL transforming growth factor-*β*3 (TGF-*β*3), and a total of 5 × 10^5^ cells at passage 3 were washed with phosphate-buffered saline, suspended in a 15 mL polypropylene tube, centrifuged at 500 g for 5 min, and cultured in chondrogenesis medium containing 100 nM dexamethasone, 100 nM sodium pyruvate, 100 nM proline 10 ng/mL transforming growth factor- *β*3, and 100 nM dexamethasone (Cyagen) for 28 days. The medium was replaced every three days. For microscopy, the pellets were embedded in paraffin, cut into 4 *μ*m sections, and stained with Alcian blue (Sigma Aldrich).

### 2.7. Determination of the Relative Secretion Levels of Human Angiogenesis-Related Proteins

Human angiogenesis-related factors secreted by SF-MSCs were measured by using a Proteome Profiler™ Human XL Cytokine Array Kit (R&D Systems). In detail, 2 × 10^6^ cells at passage 3 were washed with PBS and incubated with 10 mL serum-free DMEM in a 10 cm dish for 24 h (*n* = 3). The medium was harvested and lyophilized for detection. We used special reagents containing 55 angiogenesis-related antibodies spotted in duplicate on nitrocellulose membranes bound to specific target proteins present in the sample. The captured proteins were detected with biotinylated detection antibodies and then visualized using chemiluminescent detection reagents. Then, the membranes were exposed to X-ray film for 10 min. We use the Quantity One software to record the film on the diaphragm site display and classified the factors according to the reference list provided. Bone marrow mesenchymal stem cells (BM-MSCs) served as a control.

### 2.8. Histology of Popliteal Cyst Wall Tissue

Cyst wall tissue was reserved after the cyst fluid was extracted. Popliteal cyst wall tissue is exposed after popliteal cyst is cut vertically (*n* = 3).

#### 2.8.1. HE Staining and Masson Staining of the Popliteal Cyst Wall Tissue

The cyst wall tissue was washed twice with PBS and fixed in 4.0% paraformaldehyde for 1 day at 25-30°C. The samples were cut into three pieces of cyst wall tissue. All samples were embedded in paraffin and cut into 5 *μ*m thick sections that were stained with HE (Servicebio) and Masson (Servicebio). The sections were examined under a light microscope. ImageJ (NIH) was used for image analysis.

#### 2.8.2. Immunohistochemistry of the Popliteal Cyst Wall Tissue

The expression of CD31 (vein endothelial cells marker) and vWF (neovascularization marker) in the popliteal cyst inside wall tissue from the patients who underwent cystectomy was detected by immunohistochemistry. The samples were incubated with primary antibodies against CD31 (Servicebio) or vWF (Servicebio) and subsequently with horseradish peroxidase- (HRP-) conjugated secondary antibody (Servicebio). The sections were examined under a light microscope. The ImageJ (NIH) was utilized to analyze the CD31 or vWF area in the constructs or defect sites. The expression of AE1/3 (epithelial cell marker) in the popliteal cyst inside wall tissue from patients who underwent cystectomy was detected by immunohistochemistry. The samples were incubated with primary antibodies against AE1/3 (Novocastra) and subsequently with horseradish peroxidase- (HRP-) conjugated secondary antibody (Novocastra). The sections were examined under a light microscope. ImageJ (NIH) was used to analyze the AE1/3 area in the constructs or defect sites.

## 3. Results

### 3.1. Isolation and Expansion of Urine Cells In Vitro

#### 3.1.1. Cell Morphology Observation and Cell Proliferation Assays

**Figure 2:**
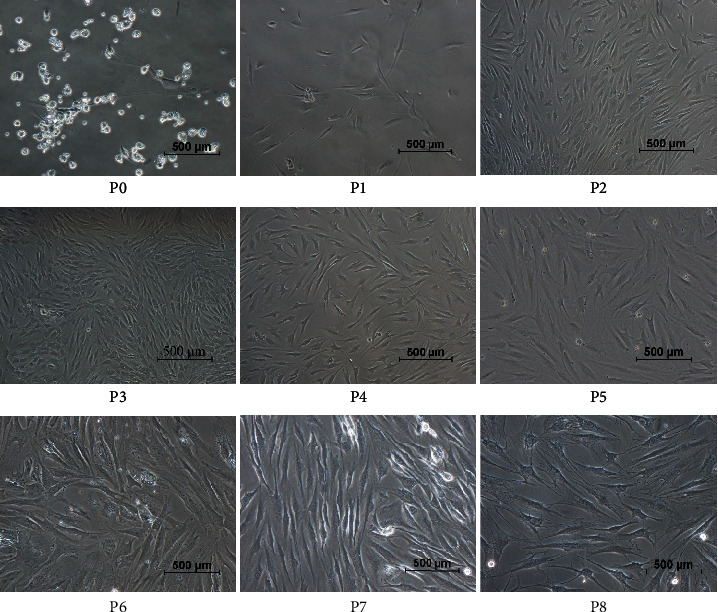
Morphological characteristics of SF-MSCs. P0: representative characteristics of SF-MSCs at primary passage within 48 h—the cell exhibiting individual colony adhered to the culture plates and nonadherent or few adherent small round cells are scattered in the culture plates (100x). P1-P4: representative characteristics of SF-MSCs from passage 1 to passage 3, between day 3 and day 10—cells exhibit the fibroblastic spindle-like shape (100x). P5-P8: representative characteristics of SF-MSCs from passage 4 to passage 8—the cells show fibrocyte-like form with long fusiform shape, grow in the same direction, and maintain the morphology (100x).

**Figure 3:**
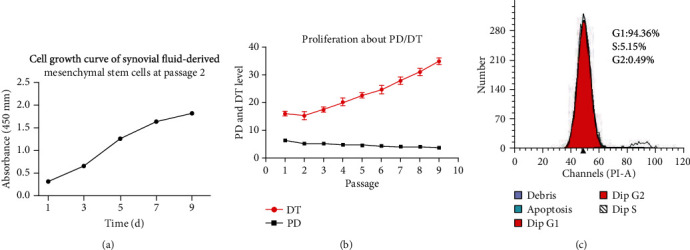
(a) SF-MSC growth curve: cells showed normal exponential cell growth patterns with a steady increase in number during an 11-day culture period. The determination and calculation of the doubling time to assess proliferation (CCK-8) showed that the growth rate was slow during the first 3 days and rapid from 4 to 8 days. The cells reached the platform stage (stopped growing or entered apoptosis) at 9~11 days. These cells showed normal exponential cell growth patterns with a steady increase in number during an 11-day culture period, as well as general mesenchymal stem cell features. (b) Curve diagram of population doublings and doubling time with passages of rUSCs. (c) Cell cycle of SF-MSCs: the data showed that 94.36% of cells were in G0/G1 phase, 0.49% were in G2/M phase, and only 5.15% were in the S phase, indicating that the cells have strong proliferative abilities.

After plating and culturing the mixed samples for 48 h, most individual cells adhered to the culture plates (P0). After the first medium change, nonadherent or few adherent small round cells in the primary culture were discarded. As shown in Figure 2, the cells exhibited a fibroblastic spindle-like shape (P1-P4). The cell colonies from passages 5 to 8 remained in a fibrocyte-like form with a long fusiform shape and grew in the same direction (P5-P8). The determination and calculation of the doubling time to assess proliferation (Cell Counting Kit-8 (Sigma-Aldrich)) showed that the growth rate was slow during the first 3 days and rapid from 4 to 8 days. The time of confluence is about the 4th day. The cells reached the platform stage (stopped growing or entered apoptosis) at 9~11 days, as shown in Figure 3(a). These cells showed normal exponential cell growth patterns with a steady increase in number during the first 9-day culture period, as well as general mesenchymal stem cell features. In addition, due to the capacity limit of the culture plate, the cells reached the platform stage. Curve diagram of calculation of the doubling time Figure 3(a) and Table 2 showed that SF-MSCs maintain relatively stable proliferation after successive passages, and the growth pattern of SF-MSCs is in accordance with characteristics of mesenchymal stem cells.

#### 3.1.2. Apoptosis Assay and Hoechst 33342 Staining

**Figure 4:**
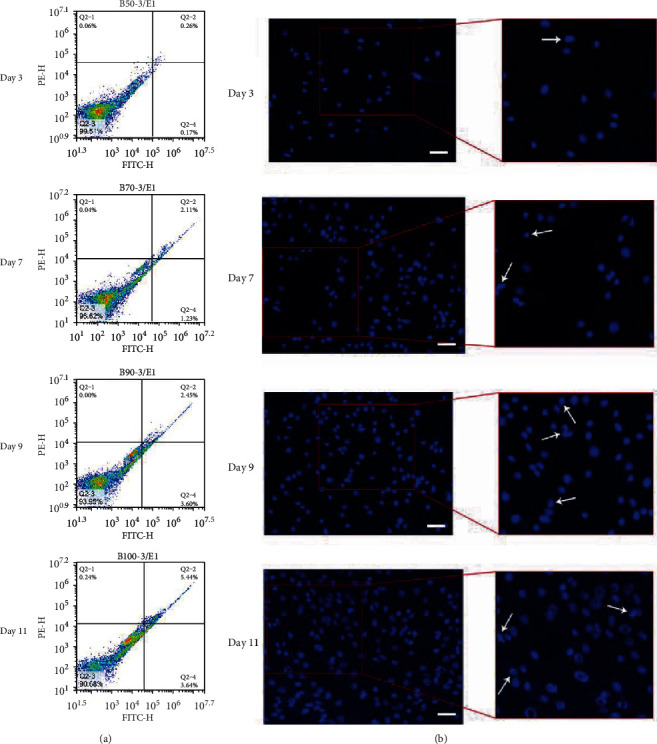
Cell apoptosis analyses of SF-MSCs after culture for 3, 7, 9, and 11 days. (a) Apoptotic cells were quantified by double-supravital staining with Annexin V-FITC and PI. (b) Cells were stained with Hoechst 33342 and were examined under a fluorescence microscope at magnification of ×200 (scale bar = 50 *μ*m). The arrows indicate several apoptotic cells with typical condensation of chromatin, cell shrinkage, and nuclear fragmentation.

Nuclear morphological changes were observed by Hoechst 33258 staining. The apoptosis of the cells was investigated using the Annexin V-FITC and PI double staining assay kit. With the increase in culture time, the apoptosis rate was correspondingly increased, and the percentage values of apoptotic cells are 0.43%, 3.34%, 6.05%, and 9.08% cultured with complete culture medium for 3, 7, 9, and 11 days, respectively (Figure 4). Moreover, the percentage values of living cells still maintained the high level. Thus, these results showed that the cell growth patterns are consistent with the cell growth curve described previously.

### 3.2. Cell Cycle Analysis

Cell cycle results were analyzed by FlowJo 10. The data showed that 94.36% of cells were in the G0/G1 phase, 0.49% were in the G2/M phase, and 5.15% were in the S phase. The data indicated that the cells had strong proliferative abilities, as shown in Figure 3(c).

PD = ln(*N*_*f*_/*N*_*i*_)/ln(2); DT = *C*_*t*_/PD. *N*_*f*_: final number of cells; *N*_*i*_: initial number of cells; *C*_*t*_: culture time.

### 3.3. Phenotypic Analysis of SF-MSCs by Flow Cytometry

**Figure 5:**
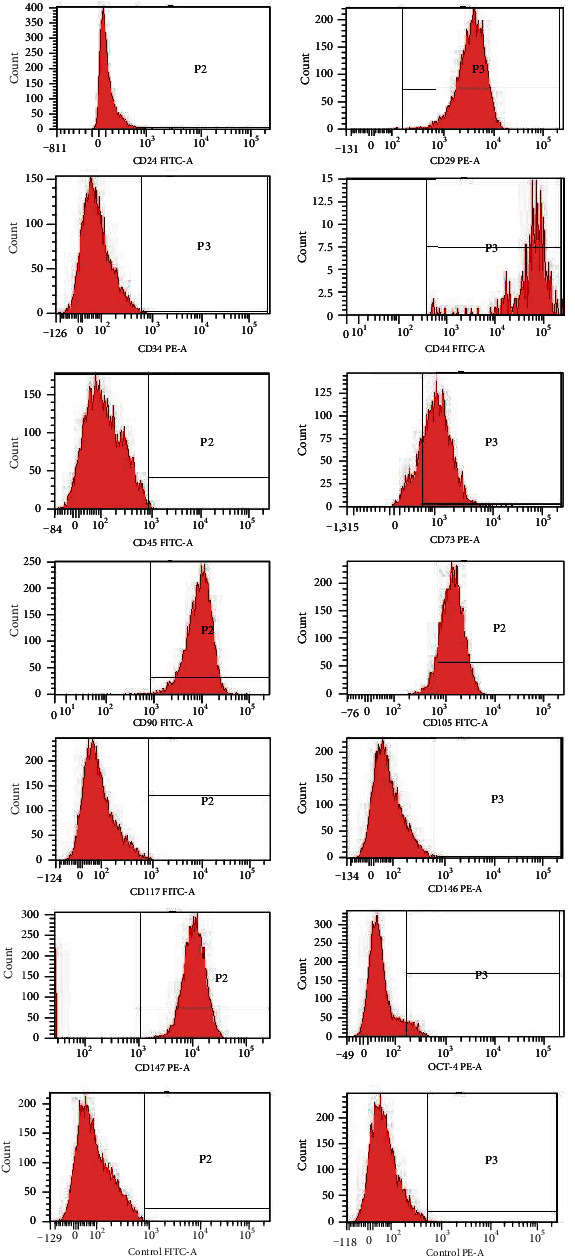
Surface marker expression of CD24, CD29, CD34, CD44, CD45, CD73, CD90, CD105, CD117, CD146, CD147, and OCT-4 in SF-MSCs after 3rd passage from the popliteal cyst analyzed by flow cytometry. Note: FITC: fluorescein isothiocyanate; PE: phycoerythrin.

Flow cytometric analysis showed that SF-MSCs highly expressed typical surface markers of MSCs (CD 29, CD44, CD73, CD90, CD105, and CD147); the embryonic stem cell surface marker OCT-4 was expressed at low levels. There was no expression of the hematopoietic cell markers CD34 and CD45. The neural stem cell surface marker CD24 and tumor cell surface markers CD117 and CD146 were not detected in our flow cytometric analysis, as shown in Figure 5 and Table 3.

### 3.4. Immunofluorescence of Different Cell Lineage Markers

**Figure 6:**
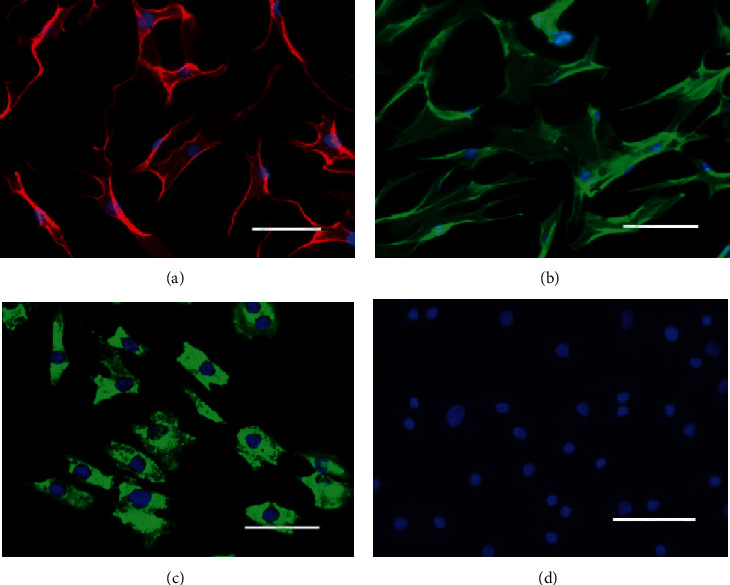
Microscopic image showing the immunofluorescent staining of SF-MSCs at passage 3. (a) Immunofluorescent staining of SF-MSCs showing positive expression of vimentin, (b) immunofluorescent staining of SF-MSCs showing positive expression of alpha-SMA, (c) immunofluorescent staining of SF-MSCs showing positive expression of collagen I, and (d) immunofluorescent staining of SF-MSCs showing negative expression of pan keratin. The nuclei were counterstained with DAPI. Scale bars = 200 *μ*m.

Using in situ immunofluorescence, we noted that SF-MSCs expressed the interstitial cell marker vimentin (Figure 6(a)), the smooth muscle cell marker alpha-SMA (Figure 6(b)), and the fibroblast-like cell marker collagen I (Figure 6(c)). None of the SF-MSCs showed the epithelial cell marker pan keratin (Figure 6(d)).

### 3.5. Multilineage Differentiation Potency of SF-MSCs

**Figure 7:**
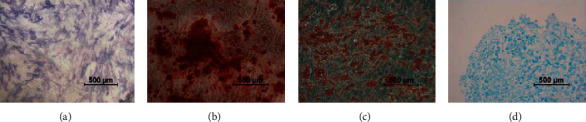
Differentiation characteristics of SF-MSCs at passage 2. (a) Representative of early osteogenesis was detected by alkaline phosphatase staining at day 7. (b) Representative of osteogenesis detected as calcium deposition is an indication of osteogenesis and was detected using Alizarin Red at day 22. (c) Representative of adipogenic differentiation visualized by Oil Red O staining of the intracellular lipid vesicles at day 21. (d) Representative of chondrogenic differentiation detected by Alcian blue staining at day 28. Micrographs are representative of 12 experiments.

Using the induced differentiation medium, we were able to differentiate SF-MSCs at passage 3 into osteocytes, adipocytes, and chondrocytes. As shown in Figure 7, SF-MSCs showed positive staining of alkaline phosphatase at day 7 (a) and calcium deposition at day 22 (b) after osteogenic differentiation. After 22-day adipogenic-induced differentiation, intracellular lipid vesicles were detected by Oil Red O staining (c). Chondrogenic differentiation was demonstrated by Alcian blue staining of the pelleted SF-MSCs at day 28, visualizing the expression of hyaluronic acid (d).

### 3.6. Angiogenesis-Related Proteins Secreted from SF-MSCs and BM-MSCs In Vitro

**Figure 8:**
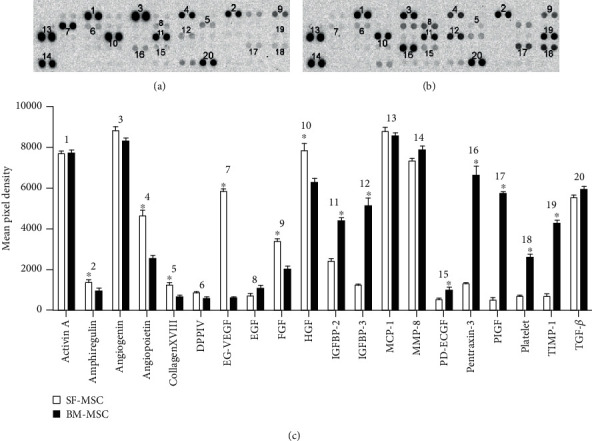
Angiogenesis-related protein growth factor secretion of SF-MSCs and BM-MSCs. (a) Result of angiogenesis growth factor secretion of SF-MSCs, (b) result of angiogenesis growth factor secretion of BM-MSCs, and (c) statistical analysis of secretion of SF-MSC and BM MSC by using Student's *t*-test: ^∗^*P* < 0.05, SF-MSC versus BM-MSC.

Twenty types of the angiogenesis-related protein such as amphiregulin, collagen XVIII, and FGF were detected in the supernatant of the culture medium of SF-MSCs and 11 in BM-MSCs (Figures 8(a) and 7(b)). The result showed that the expression of some angiogenesis-related proteins in SF-MSCs was significantly higher than that in BM-MSCs, which include amphiregulin, angiopoietin, collagen XVIII, EG-VEGF, FGF, and HGF. (Figure 8(c), Student's *t*-test: ^∗^*P* < 0.05, *n* = 3).

### 3.7. Histological Assessment of Cyst Wall Tissue

**Figure 9:**
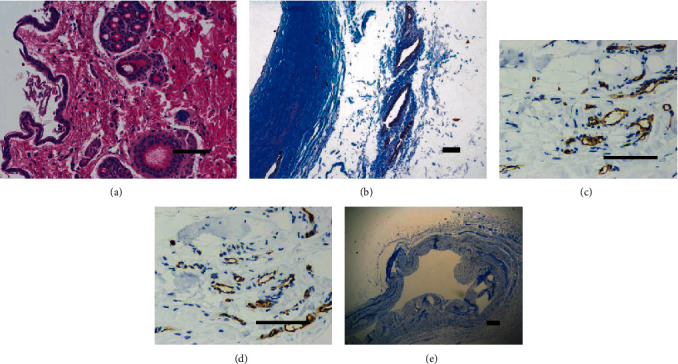
Histological assessment of cyst wall tissue. The HE staining (a) and Masson staining (b) of cyst wall tissue show the presence of endothelial cells of blood vessels in popliteal cyst. Immunohistochemical staining of CD31 (c) and vWF (d) showed positive expression. Immunohistochemical staining of inside wall tissue of popliteal cyst cavity showed negative expression of AE1/3 (e). Scale bars = 200 *μ*m.

HE staining and Masson staining of the cyst wall tissue were performed. Representative images of HE staining (Figure 9(a)) and Masson staining (Figure 9(b)) of the vascular cavity are shown to verify the presence of endothelial cells of blood vessels in the popliteal cyst. Immunohistochemical staining of CD31 (Figure 9(c)) and vWF (Figure 9(d)) showed positive expression, indicating that the popliteal cyst cavity connects with blood vessels. Immunohistochemical staining of inside wall tissue of the popliteal cyst cavity showed negative expression of AE1/3 (Figure 9(e)), indicating that there was no epithelial cell in the popliteal cyst inside the wall tissue.

## 4. Discussion

In recent years, tissue engineering has developed rapidly, and the application of stem cells has become increasingly extensive. Due to the self-renewal and multidirectional differentiation ability of stem cells, this topic has been a research hotspot. Medical applications include reconstruction of the bone, cartilage, blood vessels, nerves, skin, and urinary system, which brings hope to patients to relieve pain. According to the developmental stage of the stem cells, they are divided into embryonic and adult stem cells. Due to ethical problems, embryonic stem cell development is limited, but adult stem cells bring hope for the clinical application of stem cells. Although no studies have shown that adult stem cells can dedifferentiate and transform into embryonic stem cells under certain conditions, studies have shown that adult stem cells can differentiate into the same tissue cells as the germ layer of their origin [22]. Osteochondral injury is a common disease in the clinic. Because cartilage has no blood vessels, nutrients cannot be transported to the articular cartilage through the blood [23]. Therefore, once the mature cartilage is damaged, it cannot be regenerated. Traditional treatment methods include drugs or joint replacement. The former only relieves pain and other symptoms; the latter has the disadvantages of high surgical risk, trauma, and high cost. At present, there is no treatment for reversing the damage to and degenerative diseases of articular cartilage [24], so mesenchymal stromal cells with cartilage repair ability have become a research hotspot.

In 2001, De Bari et al. [7] successfully isolated synovial fluid-derived mesenchymal stromal cells (SF-MSCs) from human synovial tissue for the first time. In vitro, some studies have shown that SF-MSCs secrete more cartilage matrix than periosteum-, fat-, or muscle tissue-derived MSCs [25]. Other studies have confirmed that SF-MSCs have a greater ability to form cartilage than bone marrow-, fat-, or skeletal muscle-derived MSCs [26]. The traditional methods of obtaining SF-MSCs can be divided into tissue excision methods and intra-articular injection or digestion methods [27]. However, surgery causes much trauma to patients, the technology requires very complicated conditions, and there are many contraindications for surgery. The SF-MSCs in this study were obtained from the popliteal cyst cut by routine resection operation. The advantages of our methods are turning waste material into treasure, achieving the reuse of synovial fluid, minimal cell damage, and absence of enzymatic hydrolysis. There were no other impurities that needed to be digested, the cells are easy to obtain and culture, and they can be sent to the laboratory for mixing with culture just by aspirating the cyst fluid. The number of cultured cells obtained is massive; for example, 1 mL of cystic fluid provided a total of 10^7^ cells by passaging 2 times. In this study, the growth curve and cell cycle analysis of SF-MSCs isolated from the synovial cyst fluid were performed. The results showed that the SF-MSCs isolated from the synovial cyst fluid grew exponentially, and the cell cycle was consistent with that of stem cell growth. Flow cytometry results showed the expression of the synovial fluid-derived mesenchymal stem cell surface markers CD29, CD44,CD73, CD90, CD105, and CD147 but not the neural stem cell surface marker CD24 or the hematopoietic stem cell surface markers CD34 and CD45, and the cells did not express the interstitial tumor cell markers CD117 and malignant epithelial CD146. It is worth noting that the expression level of CD105 (86.5%) was high, and CD105 is a noticeable marker of articular cartilage MSCs [28]. Its high expression indicated that the cells have a strong cartilage-forming ability. CD105 is a TGF-*β*3-type receptor, also known as SH-2, which mediates the interconnection of various signaling proteins in the cytoplasm and forms a protein heteromeric complex. CD105 is involved in cell signaling by modulating the binding of TGF-*β*3 to its receptor [29]. Cellular immunofluorescence suggested that the SF-MSCs obtained from popliteal cyst fluid were positive for the mesenchymal cell marker vimentin and negative for the epithelial cell marker pan keratin, indicating that these cells originated from mesenchymal tissue but not epithelial tissue, further proving that they were consistent with the typical characteristic of SF-MSCs provided by other researchers [30]. The SF-MSCs obtained also reacted positively with antibodies against *α*-SMA and the type I collagen (collagen I), indicating that the SF-MSCs had fibroblast-like characteristics. One of the important characteristics of stem cells is the potential for multidirectional differentiation. The SF-MSCs isolated from the popliteal cyst fluid have excellent osteogenic, adipogenic, and cartilage-forming abilities, which is strong evidence for their multidirectional differentiation potential.

However, the origin and role of SF-MSCs have not yet been determined. Jones et al. [31] suggested that SF-MSCs may be derived from damaged cartilage, bone, synovium, periosteum, or bone marrow. The children with popliteal cysts included in this study did not have bone and joint diseases, and so the SF-MSCs were not from damaged bone or cartilage. Pittenger and colleagues' research [32] has demonstrated that SF-MSCs were distinct from bone marrow mesenchymal stem cells (BM-MSCs); in their result, SF-MSCs form a pool of highly clonogenic cells with chondrogenic potential, whereas BM-MSCs are very heterogeneous [33]. This fact suggested that SF-MSCs were not derived from BM-MSCs but from adjacent synovium [34]. This corroborates the findings from another study, which showed that SF-MSCs were likely to be closer to synovium MSCs than to BM-MSCs [35]. Morito et al. [36] reported that the number of SF-MSCs increased in knee lavage fluid after injury of the knee joint, which to some extent proved that SF-MSCs may originate from the damaged endometrium. Synovial tissue can be divided into two distinct anatomical levels: the intimal tissue and the subintima. The intimal tissue is loose and avascular and has no basement membrane support. The intimal layer is composed of tissues filled with cells and vascular networks. Fibroblast-like synoviocytes (FLSs) are present in the normal intima and subintima [37]. Some studies [38, 39] have confirmed that some FLSs exhibit multidirectional differentiation abilities under specific culture conditions. Based on the theory of two-layer synovial tissue, De Bari et al. [7] believe that SF-MSCs may originate from bone marrow precursor cells or perivascular cells. However, the spatial distribution of synovial stromal cells in the synovial tissue has not been elucidated yet. Harvanová et al. [40] reported that CD105-positive cells accounted for 40% to 50% of SF-MSCs digested by synoviolin and increased to 95% by immunomagnetic separation. However, our data showed that CD105 positivity by immunomagnetic beads was approximately 86.5%, which indicates that the SF-MSCs we obtained may be derived from different synovial tissue origins.

Adult popliteal cysts are mostly caused by chronic lesions caused by disease of the bursa itself and chronic knee joint disease [41]. Clinical studies have shown that the bursa of adult popliteal cysts is connected to the joint cavity, but MRI examination of clinically collected popliteal cysts in children shows that the bursa does not communicate with the joint cavity. Sun et al. [42] found that a small amount of vascular cell adhesion molecule-1 (VCAM-1) was expressed in the primary cell population in synovial fluid in patients with temporomandibular joint disorder. It is speculated that some synovial cells may be derived from deciduous fragments of the vascular wall layer and damaged circulating bone marrow mesenchymal stem cells. To explore the source of synovial fluid in the popliteal cyst, we performed HE and Masson staining tests on the vertical cystic section and discovered a brief distribution of organizational structure in the popliteal cavity. To further explore the endovascular construction of the cavity, we performed immunohistochemical detection of CD31 and vWF inside the wall slice. The results showed a positive expression, indicating that the popliteal fluid was connected to blood vessels, and so we consider that the SF-MSCs isolated from the synovial fluid may be derived from cells of the vascular wall layer or circulating medium bone marrow mesenchymal stem cells that are fragments formed by damaged cells. Moreover, we performed flow cytometry detection of cell surface markers on isolated cells and confirmed that there were synovial fluid mesenchymal stromal cells. According to the International Society for Cytotherapy, MSCs must be able to adhere to plastic material and expand when cultured in vitro. In addition, MSCs must express the surface markers CD73, CD90, and CD105 and be negative for the expression of CD45, CD34, CD14, and CD11b surface molecules [43]. As for the source of SF-MSCs, they are probably derived from synovial membrane, bone marrow, and cartilage according to Table 4 [7, 44–48]. Determination of the relative secretion levels of human angiogenesis-related proteins array showed that expression of angiogenesis-related proteins in SF-MSCs was significantly higher than that in BM-MSCs, which include amphiregulin, angiopoietin, collagen XVIII, EG-VEFG, FGF, and HGF. Among them, EG-VEGF, angiopoietin, and FGF were considered to be crucial factors in promoting angiogenesis. EG-VEGF binds to three tyrosine kinase receptors containing globulin-like domains to induce the migration of vascular endothelial cells and maintain the tubular structures, and it is one of the main factors that promote angiogenesis [49, 50]. Angiopoietin- (Ang) 1/Tie-2 is an important information pathway for regulating angiogenesis. Ang-1/Tie-2 information pathway can induce smooth muscle cells, neutrophils, and eosinophils to produce chemotactic effects, which promotes mesenchymal cell transformation into smooth muscle cells and can induce the secretion of serotonin, matrix metalloproteinases, and fibrinolysin. Makinde and Agrawal [51] found that the lack of Ang-1 and Tie-2 can lead to a significant reduction of microvessels. The fibroblast growth factor is an effective angiogenesis factor and endothelial cytokinin. It does not directly regulate angiogenesis but can significantly enhance the expression of platelet selectin and intercellular adhesion molecule 1 [52], which indirectly plays a regulatory role. Hagedorn et al. [53] found that platelet factor 4 can prevent the binding of the fibroblast growth factor with endothelial cells and the formation of their dimers, thereby inhibiting the formation of new blood vessels. It can be used as an entry point to screen antivascular drugs for the treatment vascular dependent diseases such as cancer.

To further explore the source of synovial fluid-derived mesenchymal stromal cells in the synovial fluid of the popliteal cyst, we performed immunohistochemical experiments on the AE1/3 of the sac wall, and the result was negative, indicating that the cells isolated from the synovial fluid were not the exfoliated cells of the endothelium of the popliteal cyst inside wall tissue but probably derived from exfoliated vascular endothelial cells. To sum up, we believe that the popliteal cyst fluid may be derived from cells shed from the vascular wall layer and fragments formed from damaged circulating bone marrow mesenchymal stem cells.

The root of the popliteal cyst is attached to the semimembranosus and semitendinosus tendon; the tendon sheath is composed of the outer fibrin sheath and the inner synovial sheath, and the synovial sheath is double-layered with an inside and an outside. The inner layer is tightly wrapped around the surface of the tendon; the outer layer is in close contact with the inner surface of the tendon sheath. A small amount of synovial fluid is contained between the inner and outer layers, and some children have abnormal development of the joint cavity, which causes synovial fluid secretion from the synovial sheath to be blocked and to accumulate [54–57]. Taken together, another potential source of the SF-MSCs in children's popliteal cyst fluid may be the self-renewal of the synovial membrane of the joint or the residing cells in the intima of the synovial membrane desquamating under mechanical stimulation.

## 5. Conclusions

The sources of SF-MSCs are diverse and have not yet been completely clear. In our research, we turn wasted material into treasure. A convenient and effective method for obtaining SF-MSCs was established by extracting the fluid of the popliteal cyst, and the ability of these cells to self-renew and differentiate multidirectionally was identified. Strong chondrogenic potential provides a new idea for the use of SF-MSCs in tissue repair, especially in articular cartilage repair. However, we have just detected the cell from aspects of stem cell markers; in vitro proliferation and multidirectional differentiation ability of the isolated SF-MSCs and further research on their characteristics in immune regulation and angiogenesis are needed. In brief, SF-MSCs from fluid of the popliteal cyst have the potential for self-renewal and multidirectional differentiation, especially in cartilage formation, and have stronger proliferation potential than some other sources of MSCs and may become new routine seed cells in tissue engineering in the future. However, the current research is limited to in vitro and animal experiments. How to better purify SF-MSCs and induce differentiation into target cells and ultimately serve the clinic will require further in-depth study in the future.

## Figures and Tables

**Table 1:** Profiles of synovial fluid samples obtained from the patients with popliteal cysts.

Sample no.	Age (years)	Sex (M/F)	Amount of cyst fluid (mL)	Time (day) of the first clone appearance	Number of clone 24 h after 1st clone appearance (24-well plate)	Total cell count/1 mL (P0)	Total live cell count/1 mL (P0)	Total live cell ratio (P0)
1	7	M	5	2	38	8.34 × 10^5^	6.73 × 10^5^	80.69%
2	13	F	10	3	45	9.12 × 10^5^	8.13 × 10^5^	89.14%
3	9	M	6	2.5	37	7.32 × 10^5^	6.46 × 10^5^	88.25%
4	11	F	9	2.5	41	9.35 × 10^5^	7.84 × 10^5^	83.85%
5	12	F	8	3	43	8.74 × 10^5^	6.93 × 10^5^	79.29%
6	7	M	6	2	37	10.12 × 10^5^	8.42 × 10^5^	83.20%
7	9	F	7	2	43	8.92 × 10^5^	7.53 × 10^5^	84.41%
8	5	M	4	1.5	44	7.93 × 10^5^	6.05 × 10^5^	76.29%
9	8	M	5	2	51	9.32 × 10^5^	7.23 × 10^5^	77.57%
10	7	F	6	2.5	43	8.42 × 10^5^	7.32 × 10^5^	86.94%
11	12	M	9	2	48	8.83 × 10^5^	7.14 × 10^5^	80.86%
12	6	F	5	2	41	7.34 × 10^5^	5.49 × 10^5^	74.80%
13	7	M	6	1.5	38	8.79 × 10^5^	7.32 × 10^5^	83.28%
14	9	M	6	1.5	35	8.58 × 10^5^	7.29 × 10^5^	84.96%
15	11	F	9	2	42	9.13 × 10^5^	8.07 × 10^5^	89.39%
16	8	M	8	1.5	39	9.78 × 10^5^	7.97 × 10^5^	81.49%
Mean ± SD	8.8 ± 2.3	—	6.8 ± 1.7	2.1 ± 0.5	41.6 ± 4.1	8.75 × 10^5^ ± 0.75 × 10^5^	7.2 × 10^5^ ± 0.76 × 10^5^	82.3% ± 4.3%

**Table 2:** Population doublings and doubling time with passages of SF-MSCs.

	P1	P2	P3	P4	P5	P6	P7	P8	P9
PD *n* = 6	6.28 ± 0.08	5.16 ± 0.26	5.17 ± 0.26	4.71 ± 0.28	4.35 ± 0.08	4.28 ± 0.31	4.17 ± 0.25	4.15 ± 0.33	3.82 ± 0.21
DT *n* = 6	19.1 ± 0.64	15.3 ± 1.34	17.5 ± 0.81	20.23 ± 1.17	22.72 ± 0.79	24.66 ± 1.32	27.95 ± 1.13	29.79 ± 1.99	33.55 ± 1.64

**Table 3:** FACS analysis of surface marker expression on SF-MSCs at passage 3.

CD antigens	Indicating	%
CD24	Neural stem cells	0.23 ± 0.05
CD29	Umbilical cord matrix stem cells	77.97 ± 1.35
CD34	Hematopoietic stem cells	0.17 ± 0.05
CD44	Epithelial basal cells	76.80 ± 1.84
CD45	Hematopoietic cells, fibrocytes	0.20 ± 0.08
CD73	Endothelium, epithelium	77.90 ± 3.84
CD90	Neurons, connective tissue	99.00 ± 0.54
CD105	Endothelial cells	86.00 ± 3.00
CD117	Interstitial tumor	0.10 ± 0.08
CD146	Malignant epithelial tumor	0.03 ± 0.05
CD147	Synovial membrane tissue	97.27 ± 0.65
OCT-4	Embryonic stromal cell	2.30 ± 0.70

**Table 4:** Mesenchymal stem cell markers for cells derived from bone marrow, cartilage, synovial membrane, and synovial fluid.

Tissue	Positive marker	Negative marker	Reference
Bone marrow	CD13, CD29, CD44, CD90, and CD105	CD34 and CD45	Barry and Murphy [44]
Cartilage	CD49e, Notch1, CD90, and STRO-1	Not clear	Williams et al. [45], Alsalameh et al. [46]
Synovial membrane	CD90, CD105, CD147, and CD44	CD34, CD45, CD117, and CD31	De Bari et al. [7], Sakaguchi et al. [47]
Synovial fluid	CD40, CD44, CD73, CD90, and CD 105	CD11b, CD34, and CD45	Boeuf and Richter [48]

## Data Availability

The data used to support the findings of this study are available from the corresponding author upon request.
